# Assessment of disease severity and patient prognosis in anti-GABA_B_R encephalitis with and without comorbid tumors

**DOI:** 10.3389/fneur.2023.1201324

**Published:** 2023-07-17

**Authors:** Caiyun Gao, Zhongyun Chen, Huijin Huang, Runxiu Zhu, Yingying Su

**Affiliations:** ^1^Department of Neurology, People's Hospital of Inner Mongolia Autonomous Region, Hohhot, China; ^2^Department of Neurology, Xuanwu Hospital, Capital Medical University, Beijing, China

**Keywords:** anti-gamma-aminobutyric-acid type B receptor (anti-GABA_B_R) encephalitis, tumor, clinical characteristics, prognosis, disease severity

## Abstract

**Purpose:**

This study aimed to analyze the severity of the condition and prognosis of patients with anti-gamma-aminobutyric-acid type B receptor (anti-GABA_B_R) encephalitis with tumors.

**Methods:**

Patients with anti-GABA_B_R encephalitis admitted to one of two hospitals from 2020 to 2022 were enrolled and divided into tumor and non-tumor groups. The clinical characteristics, condition severity, treatment options, and prognosis of the two groups of patients were compared and analyzed.

**Results:**

Eighteen patients with anti-GABA_B_R encephalitis were included, ten of whom had tumors. The comparison of clinical characteristics showed that rates of status epilepticus and coma were significantly higher in the group with tumors (*P* = 0.013 and *P* = 0.025, respectively); the incidences of pulmonary infection, respiratory failure, hyponatremia, and hypoproteinemia were also substantially more frequent in the tumor group (*P* = 0.054, *P* = 0.036, *P* = 0.015, and *P* = 0.025, respectively). The laboratory test result comparison showed that serum neuron-specific enolase (NSE) and carcinoembryonic antigen (CEA) were present only in the group with tumors (*P* = 0.036 and *P* = 0.092, respectively), but there was no significant difference in the occurrence of elevated CEA between the two groups. Conversely, the percentage of serum systemic autoimmune antibodies was higher in the group without tumors than in the group with tumors (*P* = 0.043). Patients with tumors tended to have poor outcomes (*P* = 0.152, OR: 7.000).

**Conclusion:**

Severe brain damage and complications occur in patients with anti-GABA_B_R encephalitis and comorbid tumors. Early screening for serum NSE and CEA helps in the early diagnosis and treatment of tumors. The prognosis is much worse for anti-GABA_B_R encephalitis with tumors.

## Introduction

In the past 10 years, as more neural autoantibodies have been discovered, an increasing number of autoimmune encephalitis (AE) cases have been identified. Anti-gamma-aminobutyric-acid type B receptor (anti-GABA_B_R) encephalitis is an autoimmune disease mediated by antibodies to GABA_B_R and was first reported in 2010 (1). The main clinical manifestations of anti-GABA_B_R encephalitis are seizures, psychiatric behaviors, and cognitive dysfunctions, accounting for 5% of all cases of AE (2). However, the risk of mortality in anti-GABA_B_R encephalitis is higher than that of other AEs, and the presence of a comorbid tumor (49.5%) is presumed to be a key contributor to mortality (3–5). Early identification of the presence of comorbid tumors is important. It was observed that comorbid tumors are mainly small-cell lung cancer and other tumor types with neuroendocrine functions. Neuron-specific enolase (NSE) is specifically located in neurons and neuroendocrine cells, so detection of NSE can be used for early screening of tumors in anti-GABA_B_R encephalitis. However, there are no studies to date on this topic.

Previous studies have been limited to the description of the phenomenon of anti-GABA_B_R encephalitis with or without tumors but have failed to sufficiently analyze the differences in clinical features and prognosis between individuals with and without tumors. Thus, in this study, we compared anti-GABA_B_R-positive patients with or without tumors in their clinical characteristics, treatment responses, and prognosis.

## Methods

### Study participants

In this retrospective study, eighteen patients with anti-GABA_B_R encephalitis were enrolled at the Department of Neurology of Xuanwu Hospital of Capital Medical University and People's Hospital of Inner Mongolia Autonomous Region between February 2020 to June 2022. The inclusion criteria were as follows. (1) Age ≥18 years. (2) Patients who met the diagnostic standards for anti-GABA_B_R encephalitis as recommended by Graus et al. (6): (a) acute or subacute onset of memory deficits, seizures, or psychiatric symptoms and unilateral or bilateral medial temporal lobe (MTL) abnormalities on T2-weighted fluid-attenuated inversion (FLAIR) MRI or ^18^F-fluoro-2-deoxy-d-glucose positron emission tomography (^18^F-FDG-PET). (b) the leukocyte count being >5/mm^3^ in cerebrospinal fluid (CSF) or the electroencephalogram (EEG) showed seizure/slow wave activity involving the temporal lobe. (c) positive levels of anti-GABA_B_R antibodies being present in the serum or CSF. (d) If one of the first two criteria is not met, the third one must be met. (e) Alternative causes are to be reasonably excluded.

### Data collection

All patients in this retrospective cohort study were divided into tumor and non-tumor groups based on tumor screenings. Demographic information, clinical features, imaging results, neurophysiological examinations, laboratory tests, tumor screenings, treatment options, and prognosis were also compared.

### Laboratory tests

All autoimmune antibodies against neuronal cell surface antigens or neurologic paraneoplastic antibodies against intracellular neuronal antigens were measured using indirect immunofluorescence tests (IIFT) (Euroimmun, Luebeck, Germany). The antigens included GABA_B_R, N-methyl-D-aspartate (NMDA) receptors, a-amino-3-hydroxy-5-methyl-4-isoxazol-propionic acid (AMPA) receptors, contactin-associated protein 2 (CASPR2), leucine-rich glioma-inactivated 1 (LGI-1), dipeptidyl-peptidase-like protein 6 (DPPX), IgLON family member 5 (IgLON5), Hu, Ri, Yo, CV2, amphiphysin, paraneoplastic antigen Ma2 (PNMA2), glutamic acid decarboxylase 65 (GAD65), and sry-related box genes (SOX1). Anti-nuclear antibodies (ANAs), anti-SSA, anti-SSB, anti-Ro-52, and anti-Scl-70 antibodies were also separately tested by IIFT and immunoblotting assays. Serum thyroglobulin antibody (Tg-Ab) and thyroid peroxidase antibody (TPO-Ab) were detected by electrochemiluminescence immunoassay.

### Tumor screening

All patients underwent tumor screening, which included chest/abdominal CT, abdominal ultrasonography, ^18^F-fluoro-2-deoxy-d-glucose positron emission temography (^18^F-FDG-PET), and tumor biomarkers. Tumor marker tests, including those for carbohydrate antigen 724, alpha-fetoprotein (AFP), NSE, serum cytokeratin 19 fragment antigen, carbohydrate antigen 125, carbohydrate antigen 199, carbohydrate antigen 153, and carcinoembryonic antigen (CEA), were measured by a commercial electrochemiluminescence assay (Roche Diagnostics, Mannheim, Germany).

### Treatments and prognostic assessment

All patients were assessed for condition severity before treatment using GCS and APACHE-2 scores. Treatment options included immunotherapy, antitumor therapy, and complication management. Immunotherapy treatments included first-line immunotherapy [corticosteroids and intravenous immunoglobulins (IVIg), plasma exchange (PLEX)], second-line immunotherapy [rituximab (RTX) and cyclophosphamide], and long-term immunotherapy [mycophenolate mofetil (MMF), RTX, azathioprine]. Antineoplastic therapies included tumor resection and chemotherapy. We interviewed participants over the telephone using the modified Rankin Scale (mRS) to estimate the prognosis; mRS of 0–2 was classified as a good prognosis, and mRS of 3–6 was classified as a poor prognosis (7).

### Statistical analysis

Statistical analyses were performed with the statistical software SPSS 22.0 (IBM Corporation, Armonk, NY, USA). Clinical data were expressed as descriptive statistics, and count data were expressed as frequencies, composition ratios, and rates. The test of variability was performed using a four-compartment or a columnar table (R × C) χ^2^, and Fisher's exact probability method was used when more than 20% of the theoretical frequencies in the four tables or columns were < 5. All statistical tests were bilateral tests, and differences were considered statistically significant at a *P*-value of < 0.05.

## Results

Eighteen patients were enrolled, with ten cases having associated tumors and eight cases without tumors. The comorbid tumors included seven cases of pulmonary malignancy, including six cases with small-cell lung cancer and one case with adenocarcinoma, one case of abnormal growth of mediastinal and supraclavicular lymph nodes of unknown location, one case of pancreatic cancer, and one case of primary fallopian tube cancer who had known tumors at the time of presentation with encephalitis. There were no significant differences in age, sex, medial temporal lobe T2/FLAIR high signal intensity, antibody titers, or EEG abnormalities between the two groups.

Clinical manifestations were compared between the tumor group and the non-tumor group (Table 1). Epilepsy, cognitive dysfunction, and psychiatric abnormalities were the most common nervous system symptoms at 94, 88, and 72%, respectively. However, the incidences of status epilepticus and decline in consciousness (GCS score ≤ 8) were significantly higher in the group with tumors than in the group without tumors (60 vs. 0%, *P* = 0.013; 50 vs. 0%, *P* = 0.036, respectively).

**Table 1:** Clinical characteristics of anti-GABA_B_R encephalitis.

**Variables**	**Total *n* = 18**	**With tumor *n* = 10**	**Without tumor *n* = 8**	***P-*value**
**Age, years**, ***n*** **(%)**				
< 60 (48–59)	10	4 (40)	6 (75)	0.188
≥.0 (60–79)	8	6 (60)	2 (25)	0.188
Gender, male/female, *n*	15/3	8/2	7/1	1.000
**Clinical symptoms**, ***n*** **(%)**				
Seizure	17 (94)	10 (100)	7 (87.5)	0.445
Status epilepticus	6 (33)	6 (60)	0 (0)	0.013
Cognitive dysfunction	16(88)	9 (90)	7 (87.5)	1.000
Psychiatric behavior	13(72)	8 (80)	5 (62.5)	0.608
Consciousness declination (GCS score ≤ 8分)	5 (27.7)	5 (50)	0	0.036
Speech disorder	3(16)	2 (20)	1 (12.5)	1.000
Complications, *n* (%)	13 (72.2)	10 (100)	3 (37.5)	0.007
Pulmonary infection	10 (55.6)	8 (80)	2 (25)	0.054
Respiratory failure	5 (27)	5 (50)	0	0.036
Hyponatremia (< 135 mmol/l)	9(50)	8 (80)	1 (12.5)	0.015
Hypoproteinemia (< 35 g/l)	8(44)	7 (70)	1 (12.5)	0.025
**Severity score**, ***n*** **(%)**				
APACHE-2 score ≥ 15	6 (33.3)	6 (60)	0 (0)	0.013
ICU admission, *n* (%)^#^	7 (38.9)	6 (60)	1 (12.5)	0.066
**CSF abnormalities**, ***n*** **(%)**				
WBC count > 5 × 10^6^/l	12 (66.7)	7 (70)	5(62.5)	1.000
Protein > 0.45 g/l	7 (38.9)	3 (30)	4(50)	0.630
Positive oligoclonal band	7 (38.9)	4 (40)	3 (37.5)	1.000
**Neuroimaging**, ***n*** **(%)**				
Medial temporal lobe T2/FLAIR hyperintensities	11 (61.1)	8 (80)	3 (37.5)	0.145
EEG abnormality, *n* (%)^*^	14 (78.8)	8 (80)	6 (75)	1.000
**Immunotherapy**, ***n*** **(%)**				
Corticosteroids or immunoglobulin only	6 (16.7)	4 (40)	2 (25)	0.638
Corticosteroids +IVIg	11 (61.1)	5 (50)	6 (75)	0.367
Corticosteroids ± IVIg+ PLEX	2 (11.1)	2 (20)	0	0.477
intravenous RTX	2 (11.1)	1 (10)	1 (12.5)	1.000
Oral mycophenolate mofetil	6 (27.8)	2 (20)	4 (50)	0.321

^*^EEG abnormalities were diffuse or focal slow waves in the interictal period, partially accompanied by paroxysmal sharp waves.

^#^Criteria for ICU admission were status epilepticus, respiratory failure (mechanical ventilation), and severe complications.

IVIg, intravenous immunoglobulin; PLEX, plasma exchange; RTX, rituximab.

The prevalence of respiratory failure, hyponatremia, and hypoproteinemia was significantly more frequent in the group with tumors than in the group without tumors (50 vs. 0%, *P* = 0.036; 80 vs. 12.5%, *P* = 0.015; 70 vs. 12.5%, *P* = 0.025, respectively). Patients with tumors were more likely to have a pulmonary infection and were admitted to the ICU, with a statistical trend (80 vs. 25%, *P* = 0.054; 60 vs. 12.5%, *P* = 0.066, respectively). The disease was more severe (APACHE-2 score ≥ 15) in those with concomitant tumors compared to those without (60% vs. 0%, *P* = 0.013) (Table 1).

Eighteen cases showed positive results for GABA_B_R antibodies in the serum, and seventeen cases had positive levels in the CSF. There was no significant difference in either high or low antibody titers when compared between groups. Serum NSE and CEA were both present in the group with tumors, and the levels were significantly different (50 vs. 0%, *P* = 0.036) or approached significance (40 vs. 0%, *P* = 0.092), respectively, when compared with the group without tumors. There was one patient who had superimposed anti-CV2 and anti-GAD65 antibodies, and one patient had anti-SOX1 antibodies in both their serum and CSF. The percentage of patients with positive serum systemic autoimmune antibodies was significantly higher in the group without tumors than in the group with tumors (62.5 vs. 10%, *P* = 0.043) (Table 2).

**Table 2:** Antibodies and tumor markers associated with anti-GABA_B_R encephalitis.

**Variables**	**Total *n* = 18**	**With tumor *n* = 10**	**Without tumor *n* = 8**	***P-*value**
**CSF**, ***n*** **(%)**				
GABA_B_R-Ab ≥ 1:100	11 (61.1)	7 (70)	4 (50)	0.630
GABA_B_R-Ab ≤ 1:32	6 (33.3)	2 (20)	4 (50)	0.321
Neurologic paraneoplastic antibodies	2 (11.1)	2 (20)	0 (0)	0.477
**Serum, n (%)**				
GABABR-Ab ≥ 1:100	11 (61.1)	7(70)	4 (50)	0.630
GABABR-Ab ≤ 1:32	7 (38.9%)	3(30)	4(50)	0.630
Neurologic paraneoplastic antibodies	2 (11.1)	2(20)	0(0)	0.477
Systemic autoimmune antibodies	6 (33.3)	1(10)	5(62.5)	0.043
NSE > 17 ng/ml	5 (27.8)	5 (50)	0 (0)	0.036
CEA > 5 ng/ml	4 (22.2)	4 (40)	0 (0)	0.092
NSE > 17 ng/ml + CEA > 5 ng/ml	3 (16.7)	3 (30)	0 (0)	0.216

CSF, cerebrospinal fluid; NSE, neuron-specific enolase; CEA, carcinoembryonic antigen.

All patients received first-line treatment in the acute phase. Of these patients, 3/18 (16.7%) received a single corticosteroid, 3/18 (16.7%) received a single IVIg, 11/18 (61.1%) received a corticosteroid combined with IVIg, and 2/18 (11.1%) received PLEX after receiving a corticosteroid or corticosteroid combined with IVIg. The two groups did not receive significantly different first-line treatments from each other. A total of 8/18 (44.4%) patients received second-line treatment in the acute phase. Two patients received RTX, and six patients received MMF, and second-line treatments were not significantly different between the two groups. Of the patients in the concomitant tumor group, three were treated with tumor resection, two with chemotherapy, four were under conservative observation, and one abandoned treatment and was discharged.

**Figure 1:**
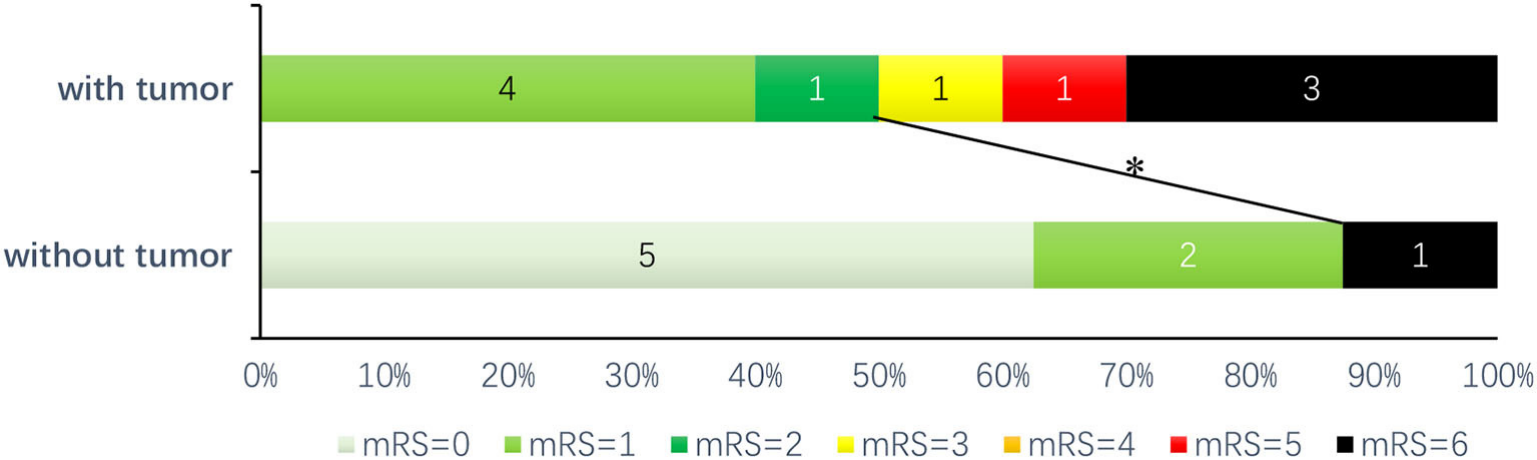
Prognosis of patients with anti-GABA_B_R encephalitis: after follow-up (1–24 months, median 8 months), ^*^there was a trend toward the good prognosis group (mRS score of 0–2) compared with the poor prognosis group (mRS score of 3–6) (*P* = 0.152, OR = 7.000, 95% CI: 0.613–79.871).

With a follow-up interval of 1–24 months (median 8 months), there was no significant difference in the prevalence of poor outcomes (mRS score of 3–6) compared to that of good outcomes (mRS score of 0–2) but showed an increasing trend (50 vs. 12.5%, *p* = 0.152). The prognosis was poorer in the group with tumors than in the group without tumors (OR 7.000, 95% CI 0.613–79.871) (Figure 1). There were four deaths among all the patients, including three patients with tumors and one without a tumor; of the patients with tumors, one died of refractory status epilepticus and two died of lung malignancy; the cause of death was unknown for the patient in the group without tumors.

## Discussion

The main findings of this study were as follows: (1) There was a high rate of comorbid tumors in anti-GABA_B_R encephalitis (55.6%), including one case of primary fallopian tube cancer detected for the first time. (2) Elevated serum NSE and CEA levels suggested anti-GABA_B_R encephalitis with potential tumors; a positive level of serum systemic autoimmune antibodies was more common in anti-GABA_B_R encephalitis without associated tumors. (3) Anti-GABA_B_R encephalitis in the presence of a tumor had a rapid onset and was severe, mainly manifesting as status epilepticus and a decline in consciousness. In addition, it also showed a high rate of complications (72.2%), such as pulmonary infections, respiratory failure, hypoproteinemia, and hyponatremia, which aggravated the severity of anti-GABA_B_R encephalitis in patients with tumors. (4) The risk of a poor prognosis was significantly increased in anti-GABA_B_R encephalitis with comorbid tumors even though patients received first- and second-line therapy.

Once AE is combined with an associated tumor, it is typically called paraneoplastic AE (8). The neurological symptoms of anti-GABA_B_R encephalitis are mainly short-term memory impairment, abnormal psychiatric behavior, and seizures, and EEG and imaging results show MTL involvement. Therefore, anti-GABA_B_R encephalitis with associated tumors is also called paraneoplastic limbic encephalitis. Status epilepticus (60%) and a decline in consciousness (GCS score ≤ 8) (50%) were more prominent in paraneoplastic anti-GABA_B_R encephalitis and were the main reasons for admission to the ICU (60%). It has been reported in the literature that the rate of status epilepticus in anti-GABA_B_R encephalitis is as high as 62%, of which 68% of cases are refractory status epilepticus; the overall rate of declining consciousness in this previous study was 51%, but no comparative analysis was performed on patients with and without tumors (9, 10). In addition, paraneoplastic anti-GABA_B_R encephalitis showed a high complication rate (72.2%) dominated by pulmonary infections (80% of complications), which was significantly higher than that of previous reports (2/3 of patients with pulmonary infection) (3). It is hypothesized that the high incidence of pulmonary infections may be related to obstructive pneumonia caused by pulmonary malignancies, hypostatic pneumonia resulting from status epilepticus and consciousness disorder, and increased risk of pneumonia opportunistic infections due to immunotherapy. Undoubtedly, severe pulmonary infections exacerbate and accelerate respiratory failure due to central hypoventilation in paraneoplastic anti-GABA_B_R encephalitis. The high occurrence of respiratory failure (50%) is another major reason for critical illness and admission to the ICU. Tumor cells have been shown to secrete antidiuretic hormones *in vivo*, leading to hypo-osmolar hyponatremia, which not only aggravates the condition but also predicts the possibility of tumors (11). The incidence of hyponatremia in paraneoplastic anti-GABA_B_R encephalitis was as high as 80%. In fact, under the double effect of tumor malignancy and lung infection, protein catabolism is greater than anabolism, leading to a 70% prevalence of hypoproteinemia. In conclusion, anti-GABA_B_R encephalitis with tumors is typically very severe and requires high levels of attention. In the acute stage, in addition to aggressive and effective immunotherapy, screening for tumors is needed.

One finding of this study was that paraneoplastic anti-GABA_B_R encephalitis had a poor prognosis with a mortality rate of 30%, similar to the previously reported mortality rate (23.2 or 41.7%), despite standardized first-line immunotherapy and antitumor therapy in the acute phase (10, 12). The reasons for this high mortality rate may be related to the severity and complications associated with the condition, poor responses to immunotherapy, and neoplasms with less differentiation. In contrast, patients who had anti-GABA_B_R encephalitis without tumors had a high prevalence of positive levels of serum systemic autoimmune antibodies (5/8), such as Tg-Ab and anti-Ro-52 antibodies (3/5). Other autoimmune diseases (ADs), such as Hashimoto's thyroiditis, Sjogren's syndrome, and systemic lupus erythematosus (SLE), in combination with anti-GABA_B_R encephalitis, have been reported (13). SLE had the highest percentage of positive anti-GABA_B_R antibodies (20.5%) (14). Although the mechanism for the coexistence of two or more antibodies is not clear, it may be related to ADs having common genetic loci that increase the risk of one AD causing another (15). This study also found that the prognosis of anti-GABA_B_R encephalitis without a tumor with two or more immune antibodies was better than that of paraneoplastic anti-GABA_B_R encephalitis; however, this finding has yet to be confirmed by a large sample and scientifically explained.

Improving the prognosis of paraneoplastic anti-GABA_B_R encephalitis is apparently challenging as it is difficult to detect tumors at an early stage. It has been reported in the literature that anti-GABA_B_R encephalitis with tumors accounts for 50% of anti-GABA_B_R encephalitis (1, 16–18). Tumors are predominantly small-cell lung cancers (SCLCs) (91.3%) as well as others such as thymoma, melanoma, rectal cancer, gastric cancer, and pancreatic cancer (4, 10, 19). Therefore, imaging and screening for tumor biomarkers in cases of anti-GABA_B_R encephalitis have become important steps for early diagnosis. This study and previous studies have found that paraneoplastic anti-GABA_B_R encephalitis has a high rate of positive serum levels of CEA and NSE (40–50%), and a positive test result motivates the further search for tumors *in vivo* (5). NSE is a macromolecular protein. CEA is an acidic glycoprotein of the human embryonic antigen-specific determinant cluster that is highly expressed in tumor and embryonic tissues. Most tumors associated with anti-GABA_B_R encephalitis, such as SCLC, have neuroendocrine attributes. SCLC usually has high expression of NSE (68.3%) or CEA (45.5%); the sensitivities of NSE and CEA in diagnosing SCLC alone are 68.3 and 45.5%, respectively, while the sensitivity of their combination increases to 81.2%; therefore, it can provide a basis for tumor screening and early diagnosis (20).

Another focus for a better prognosis in paraneoplastic anti-GABA_B_R encephalitis is to improve immunotherapy protocols. Immunotherapy regimens were similar in both groups, and PLEX was rarely used (20% of the cases included in this study), especially when the response to immunotherapy was poor. Whether enhanced immunotherapy or PLEX is preferred is not known.

### A new neoplastic category associated with anti-GABA_*B*_R encephalitis

In this study, for the first time, we found a patient (60 years old) exhibiting a case of anti-GABA_B_R encephalitis with primary fallopian tube cancer who had undergone total hysterectomy and bilateral adnexal resection 3 months before the disease and received regular chemotherapy with paclitaxel in conjunction with carboplatin after the surgery.

The pathological staging of primary tubal carcinoma is endometrioid adenocarcinoma with no carcinoma of the uterus, ovaries, abdominal lymph nodes, or omental tissue.

With status epilepticus as the initial symptom, T2/FLAIR showed a high signal in the hippocampus bilaterally and positive anti-GABA_B_R antibodies in serum and CSF tests. Death from acute pulpitis and septic shock occurred after 8 months of treatment with methylprednisolone combined with IVIg. Primary tubal cancer is a rare gynecologic malignancy, with an incidence rate of 1.0–2.0% among them (21). There have been no reports of anti-GABA_B_R encephalitis with primary tubal cancer. In theory, endometrioid adenocarcinoma with a neuroendocrine nature can express GABA receptors, and immunosurveillance against tumors may induce anti-GABABR encephalitis.

## Conclusion

In summary, there were differences in condition severity and prognosis between patients with anti-GABA_B_R encephalitis with tumors and without tumors. Hope for reducing severity lies in intensive immunotherapy and effective treatment of comorbidities. Improvements in prognosis are likely to depend on enhanced tumor screening, including biomarker testing and imaging, to provide opportunities for early diagnosis and treatment of tumors. In addition, screening for serum systemic autoimmune antibodies is important for predicting an accurate prognosis and outcome. The main limitation of this study is that the sample size was small, and the single-factor analysis alone was far from sufficient. It is expected that data will be accumulated from more cases and analyzed in a progressive stratified manner.

## Data availability statement

The original contributions presented in the study are included in the article/supplementary material, further inquiries can be directed to the corresponding author.

## Ethics statement

Ethical review and approval was not required for the study on human participants in accordance with the local legislation and institutional requirements. Written informed consent from the patients/participants or patients/participants' legal guardian/next of kin was not required to participate in this study in accordance with the national legislation and the institutional requirements.

## Author contributions

CG designed and administrated the study, analyzed data, and drafted paper. ZC and HH participated within the investigation, data curation, and analysis. RZ took part in investigation. YS provided the resources, supervised the study, and reviewed the manuscript. All authors contributed to the article and approved the submitted version.
